# Single-cell derived tumor organoids display diversity in HLA class I peptide presentation

**DOI:** 10.1038/s41467-020-19142-9

**Published:** 2020-10-21

**Authors:** Laura C. Demmers, Kai Kretzschmar, Arne Van Hoeck, Yotam E. Bar-Epraïm, Henk W. P. van den Toorn, Mandy Koomen, Gijs van Son, Joost van Gorp, Apollo Pronk, Niels Smakman, Edwin Cuppen, Hans Clevers, Albert J. R. Heck, Wei Wu

**Affiliations:** 1 Biomolecular Mass Spectrometry and Proteomics, Bijvoet Center for Biomolecular Research and Utrecht Institute for Pharmaceutical Sciences, Utrecht University, Padualaan 8, 3584 CH Utrecht, The Netherlands; 2 Netherlands Proteomics Centre, Padualaan 8, 3584 CH Utrecht, The Netherlands; 3 Oncode Institute, Hubrecht Institute, 3584 CT Utrecht, The Netherlands; 4 Hubrecht Institute, Royal Netherlands Academy of Arts and Sciences and University Medical Centre Utrecht, 3584 CT Utrecht, The Netherlands; 5 Center for Molecular Medicine and Oncode Institute, University Medical Center Utrecht, Universiteitsweg 100, 3584 CG Utrecht, The Netherlands; 6 Department of Pathology, St. Antonius Hospital, 3543 AZ Utrecht, The Netherlands; 7 Department of Surgery, Diakonessenhuis Hospital, 3582 KE Utrecht, The Netherlands; 8 Hartwig Medical Foundation, 1098 XH Amsterdam, The Netherlands; 9 Princess Máxima Center for Pediatric Oncology, 3584 CS Utrecht, The Netherlands

**Keywords:** Immunotherapy, Tumour heterogeneity

## Abstract

Tumor heterogeneity is a major cause of therapeutic resistance. Immunotherapy may exploit alternative vulnerabilities of drug-resistant cells, where tumor-specific human leukocyte antigen (HLA) peptide ligands are promising leads to invoke targeted anti-tumor responses. Here, we investigate the variability in HLA class I peptide presentation between different clonal cells of the same colorectal cancer patient, using an organoid system. While clone-specific differences in HLA peptide presentation were observed, broad inter-clone variability was even more prevalent (15–25%). By coupling organoid proteomics and HLA peptide ligandomics, we also found that tumor-specific ligands from DNA damage control and tumor suppressor source proteins were prominently presented by tumor cells, coinciding likely with the silencing of such cytoprotective functions. Collectively, these data illustrate the heterogeneous HLA peptide presentation landscape even within one individual, and hint that a multi-peptide vaccination approach against highly conserved tumor suppressors may be a viable option in patients with low tumor-mutational burden.

## Abstract (web summary)

Immunotherapy may exploit alternative vulnerabilities of drug resistant cells. Here, the authors show that the HLA peptide presentation landscape is heterogeneous even within one individual, hinting that a multi-peptide vaccination approach against highly conserved tumor suppressors may be needed.

## Introduction

Clonal diversity and tumor evolution are key drivers of cancer progression. As individual cancer cells within the same tumor tissue can divide and independently acquire beneficial genetic and epigenetic traits, the tumor becomes increasingly heterogeneous and diversely adapted to survive chemotoxic stress. As such, differential resistance to treatment is most often attributed to molecular signatures that are tumor clone specific^1,2^. Immunotherapy may then be administered in combination to eliminate residual disease^3^.

Central to the concept of such a sequential or combinatorial therapeutic regime, is shifting the treatment focus away from the “selection” of a chemo-resistant phenotype^4–6^, towards exploiting unique immune vulnerabilities in drug-resistant tumor clones^7,8^.

Immunotherapy relies on the patient’s own immune system to induce an anti-tumor response. Next to checkpoint blockade inhibitors, monoclonal antibodies and anti-tumor vaccines may be raised against tumor cells^9^ or tumor-specific cell-surface antigens (HLA class I peptide ligands), which arise as proteasome-generated byproducts from protein homeostasis^10^. While HLA class I peptide ligand presentation mirrors the “health-status” of each cell, the immense repertoire of tumor-surface peptide antigens has become a rich source of inspiration for rational design of highly personalized peptide vaccines^11–15^.

One of the key questions that still need to be addressed, is the extent of heterogeneity in HLA class I peptide ligand presentation, between tumor cells in the same environment or individual. This is important, because if substantial heterogeneity also exists at the level of tumor cell-surface ligand presentation, tumor cells that present less to the immune system could also evade from the efficacy of monoclonal antibodies and anti-tumor vaccines, just like drug-resistant cancer cells can evade from pharmacological inhibition.

In the growing field of immunopeptidomics, we and others have contributed to drastically improve HLA peptide ligand detection, through enhanced ligand isolation, preparation^16,17^, chromatographic separation^16,18^, the use of improved mass spectrometry-based peptide fragmentation/sequencing strategies^18^ and bioinformatics^19^. Despite these notable advances in analytics, it remains difficult to probe for variation in HLA class I peptide ligand presentation in tumor clonal sub-populations, or even ideally in single tumor cells. The major bottleneck remains the combination of the extreme low abundance in neo-antigens^20^ and the lack of “PCR-equivalents” for proteins and HLA peptides. Here, we amplified single-cell patient material into clonal organoids for deeper clonal proteome and HLA class I peptide ligand analyses, starting from a low mutational burden microsatellite-stable (MSS) colorectal cancer (CRC) patient harboring the HLA-A*02:01, HLA-B*15:01/57:01, and HLA-C*03:04/06:02 alleles. Organoids grown in controlled culture are known to be genetically stable, and should retain the molecular signatures and surface marks of the originating cells^21–23^, making the organoid technology in our investigation an ideal system to also amplify the protein and HLA class I peptide ligands.

Using this approach, we detected clear inter clonal proteome and HLA ligandome heterogeneity, detecting a large amount of HLA class I peptide ligands (about 7000) across four different colorectal cancer clones, that had been isolated concurrently from the same individual patient in vivo, and also amplified using the organoid technology under the same in vitro conditions. Comparing against peptide ligands presented by normal colon organoids, also from the same donor, about 300 HLA class I peptide ligands were presented exclusively by the tumor clones. We further identified a considerable number of clone-specific antigens and tumor-shared antigens originating from several notorious oncogenic proteins. Interestingly, these unique peptide ligand signatures were largely uncoupled from the trends observed at the proteome level within the same clones, exemplifying again current limits in predicting antigen presentation based on DNA, RNA, or protein-level regulation.

To the best of our knowledge, this is the first investigation on intra-patient clonal diversity in HLA class I peptide presentation. The findings we distill here should ultimately contribute towards improving therapeutic considerations and efficacy in personalized immunotherapy.

## Results

### Single-cell models of colorectal cancer by using organoid amplification

**Fig. 1:**
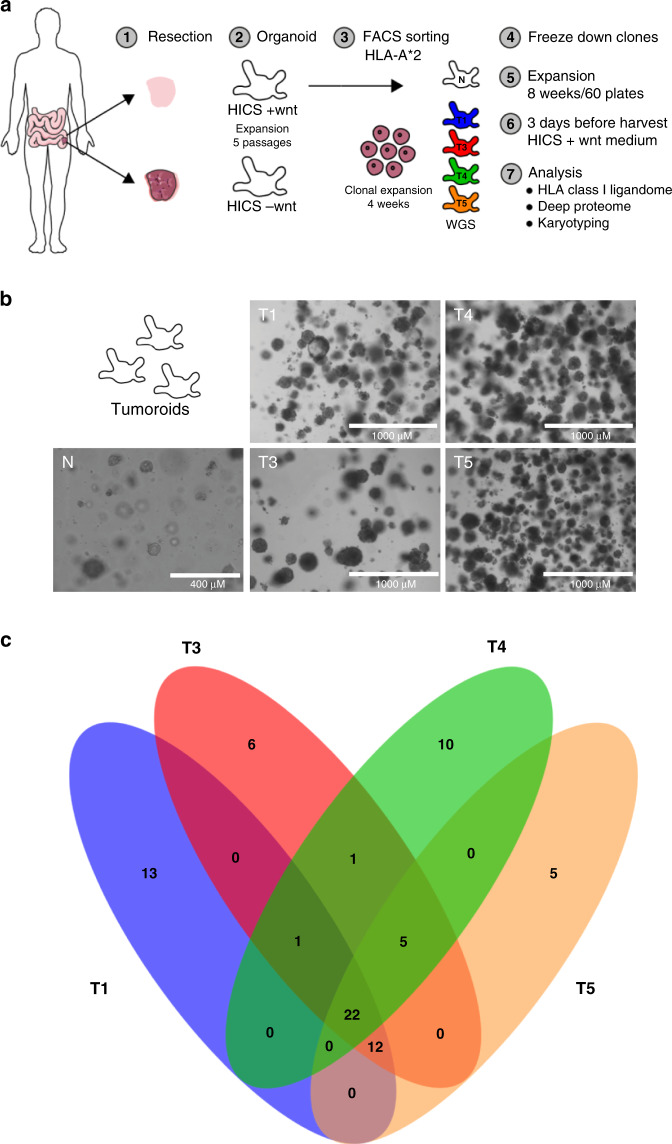
Preparation and characterization of single-cell derived clonal colorectal organoids. **a** Organoids were made from colorectal cancer tissue and healthy tissue from the same CRC patient and grown in human intestinal stem cell medium (HICS) with or without additional wnt. After five passages, the cells were FACS sorted for HLA-A*2 positive cells and were then clonally expanded for four weeks. The clones were frozen and expanded again for approximately eight weeks until 60 plates. Three days before harvest, the tumor clones were cultured in HICS +wnt medium. **b** Representative images of organoid morphology from 3 different passages. Four CRC tumor organoid lines (T1, T3, T4, and T5) were used together with a normal CRC organoid line (N). Magnification indicated by respective scale bars. **c** Venn diagram showing the shared and unique somatic mutations (SBS and INDELs) of each CRC organoid line.

Our first objective was to generate patient-derived organoid clones, from single tumor cells that retain heterogeneity and recapitulate the hallmarks of CRC (schematic, Fig. 1a). From the same patient, four tumor clones were isolated and maintained in organoid culture, alongside a normal colon organoid line generated from tumor-free colon mucosal tissue biopsied from the same patient. As shown in Fig. 1b, the tumor clones T1, T3, T4, and T5 are morphologically distinct from normal organoids of the same patient. Somatic mutation analysis of each CRC organoid clone, against the normal colon organoid line as germline reference, revealed that many exonic mutations are shared between the CRC clones (Fig. 1c and Supplementary Fig. 1). However, every CRC organoid clone also harbored unique mutations that recapitulate intratumor heterogeneity and reveal that the tumor clones are not genetically identical. DNA copy number analysis also revealed a conserved duplication of chromosome 7 and 8 and chromosomal arm 13q (Supplementary Fig. 1), which are established characteristics for CRC^24^.

### Single-cell amplified CRC organoid proteomes reveal clonal heterogeneity

**Fig. 2:**
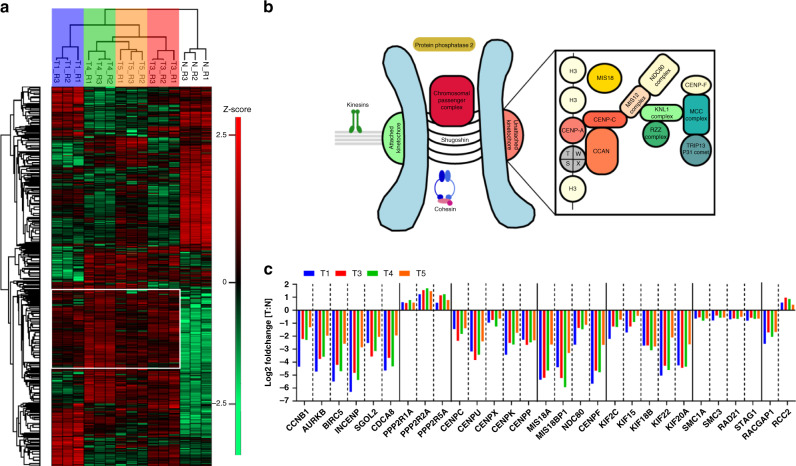
CRC organoids recapitulate tumor proteome characteristics and retain clonal heterogeneity. **a** Clustering of ANOVA significant proteins (*p* < 0.05). A conserved tumor proteome characteristic signature is indicated by the white box, whereas pockets of clonal heterogeneity are also observed in each single-cell derived tumor organoid line. **b** Schematic representation of the kinetochore and associated protein complexes upon unattached microtubules. Numerous proteins in the kinetochore regulatory complex were significantly reduced in steady-state abundance. **c** Proteome level fold change analysis revealed consistent loss of chromosomal regulatory proteins in every tumor clone compared to normal colon organoids. Plotting data tabulated in Source Data.

**Fig. 3:**
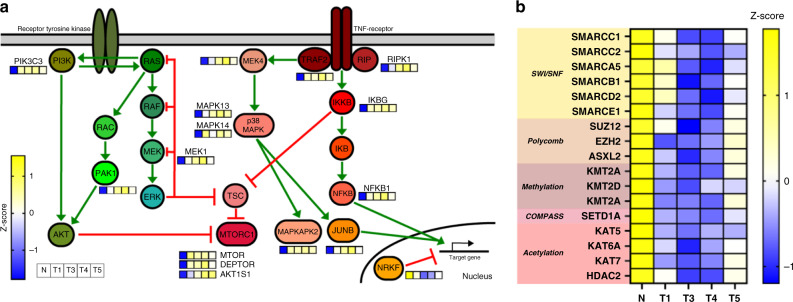
Proteome characteristics retained in clonal heterogeneity. **a** Schematic map of the mTOR pathway featuring proteins detected in the deep proteome analysis. Z-scored protein intensities are indicated in small squares. Collectively, the tumor organoid clones have higher steady-state abundance of mTOR proteins when compared to the normal organoid line. **b** Heatmap of Z-scored protein abundances of chromatin remodeling complex components and various transferases, demethylases, and deacetylases. Compensatory differences in protein abundance within the same functional processes illustrate heterogeneity between CRC organoid lines. Plotting data tabulated in Source Data.

Next to somatic mutation analysis of each CRC organoid clone, we further compared the tumor clones by deep label-free quantitative proteomics, with abundance comparisons made across ~6000 proteins. As shown in Fig. 2a, proteome signatures of tumor clones were noticeably different from the paired normal organoid line. Cluster analysis in Fig. 2a reveals proteome characteristics shared by all four tumor clones, which includes numerous proteins involved in chromosomal segregation (Fig. 2b, c) and mTOR signaling (Fig. 3a). Compared to the normal clone, a statistically significant decrease in numerous proteins that mediate mitotic spindle assembly, regulate spindle assembly checkpoint^25,26^, or form the chromosomal passenger complex^27^ (schematic in Fig. 2b) was observed. The protein-level downregulation of these key regulators of chromosomal integrity was found to be highly consistent across all four tumor organoid lines, with respect to the normal control (Fig. 2c), for instance, the downregulation of Aurora B (up to 25-fold), Survivin (up to 45-fold) and INCENP (up to 78-fold). Phenotypic defects in chromosomal segregation in these CRC organoids were further corroborated by subsequent karyotyping analysis. Karyotype analysis of the end-stage organoid clones revealed aneuploidy in all four CRC clones, with >50% of cells analyzed having a chromosome number outside the normal range of 44–46 (Supplementary Fig. 2). This is consistent with aneuploidy documented in the majority of solid tumors^28^. The differential degree of aneuploidy between the tumor clones on the other hand also demonstrates diversity in chromosomal aberrations between the tumor clones, likely pre-existing at the point of isolation.

In addition, components in mTOR signaling also appeared to be consistently regulated in all tumor clones with respect to the normal organoid clone (Fig. 3a). Uncontrolled mTOR signaling for proliferation is a well-documented functionality required to support CRC oncogenesis^29^, and our proteome level observations reflect the known activation of mTOR pathway in CRC pathogenesis. Based on these evidence, the CRC tumor clones isolated exemplify the hallmarks of chromosomally instable CRC, and are good single-cell models to study intrinsic micro-heterogeneity in (i) proteome regulation and (ii) HLA class I peptide ligand presentation.

Despite substantial convergence, individual tumor clones were nonetheless not identical, as pockets of clone-specific proteome signatures were prominently detected. These clone-specific differences in general represent redundant hits in the same functional pathway that could modulate, replace, or rescue one other. For instance, some clones (notably T1, T3, and T4) were hallmarked by the collective loss of DNA-level regulation through overall reduction in the capacity for methylation and acetylation turnover, but mosaic suppression on KMT2A/KMT2D/KDM2A and KAT5/KAT6A/KAT7/HDAC2 (Fig. 3c). Substantial dysregulation of chromatin remodeling complexes and epigenetic regulation was found to be also a striking signature for some of the clones (notably T3 and T4), and has also been documented as a hallmark of CRC and numerous other cancer types^30–33^. Taken together, these findings demonstrate that although all four tumor clones were isolated from the same patient, substantial heterogeneity still exists between these clones, especially where there is functional redundancy. Since such heterogeneity still exists after in vitro organoid amplification in the same controlled environment, these differences are most likely to be intrinsic or imprinted in vivo, in agreement with prior report that clone-specific differences are largely preserved with organoid amplification^21^.

### HLA class I peptide ligand analysis from clonal organoids

To address if the observed heterogeneity also exists in antigen presentation between tumor organoid clones of the same individual, we isolated HLA class I peptide ligands from each tumor clone by immuno-affinity purification with a pan-HLA class I antibody (W6/32). Peptide LC-MS/MS analyses with complementary HCD and EThcD fragmentation modes (experiments 1 and 2, respectively) enabled high-sensitivity detection of a large number of peptide ligands (approximately 7000) from each tumor clone (Supplementary Fig. 3a). In addition, at least 85% of all peptides identified were predicted to bind to the patient’s HLA type (HLA-A*02:01, HLA-B*15:01/57:01, HLA-C*03:04/06:02) (Supplementary Fig. 3b). The organoid-derived peptide ligands were also predominantly 9 amino acids long (Supplementary Fig. 3c), and in good concordance with the theoretical HLA-type specific peptide consensus motifs (Supplementary Fig. 3d). Collectively, these data confirm the high quality and specificity in our ligandome sample preparation and data analysis.

**Fig. 4:**
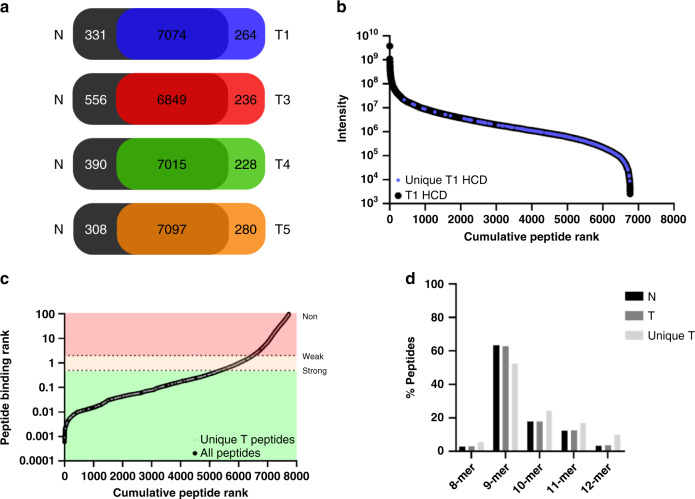
Tumor-specific HLA class I peptide ligand characteristics. **a** Identification overlap in unique HLA peptide ligands between normal CRC organoids and each tumor organoid line. In all comparisons, the majority of HLA peptide ligands (>6800) were presented by both normal colon organoids and tumor organoid clones. **b** Peptide intensities plotted against the cumulative peptide rank. HLA peptide ligands identified from T1 were ranked by decreasing intensity. On the representative trace of all T1 – HCD peptides (black), peptides that are unique to T1 and never detected from normal colon organoids are annotated (blue). The spread of these T1 unique peptides over ranked intensity indicated that several T1 unique peptides are highly abundant. **c** Peptide binding rank plotted against cumulative peptide rank. Peptide binding ranks were predicted using the NetMHC 4.0 pan algorithm. Peptides with a binding rank below 0.5 are considered strong binder. Peptides with a binding rank between 0.5 and 2.0 are considered weak binders and all peptides with a binding rank over 2.0 are considered non-binders. On the trace of all peptides identified (black), tumor-specific peptides that are never detected from the normal colon organoids (in 6 MS measurements by 2 different fragmentation methods) are annotated (gray). Distribution of peptides presented uniquely by tumor organoid clones reveals many tumor-specific ligands with high affinity. **d** Length distribution of peptide ligands from normal (N), tumor (T), and unique tumor (T unique). Peptide ligands unique to CRC tumor organoids appear to be spread over a broader length distribution. Ligand characteristics for each individual tumor organoid clone are shown in Figs. S1 and S2. Plotting data tabulated in Source Data.

Since the CRC clonal proteomes were easily markedly distinguishable from the normal organoids by various conserved signatures (Fig. 2a), we hypothesized that corroborating differences in the ligands presented should be detectable, as HLA peptide ligands are known to originate as byproducts of protein turnover. To our surprise, only about 3% of all HLA peptide ligands detected were unique to each CRC tumor clone, and never presented by the normal organoids (Fig. 4a). By ranking the peptide intensities (label-free quantification) and binding affinities of all ligands detected from each tumor clones, we found that clone-specific ligands were not necessarily always low abundant (Fig. 4b, Supplementary Fig. 4) or low in peptide loading affinity to the patients HLA type (Fig. 4c). Rather, these provide indications that abundant and high-affinity tumor-specific HLA class I peptide ligands could still be present in the small proportion of tumor-unique presented peptides, despite the low mutation load. In addition, we also observed a slight re-distribution in HLA class I peptide ligand length away from 9-mers (Fig. 4d), potentially hinting at modulations in the HLA peptide trimming mechanism in the tumor cells.

### Clonal HLA class I ligandomes display over-representation of DNA repair source proteins

**Fig. 5:**
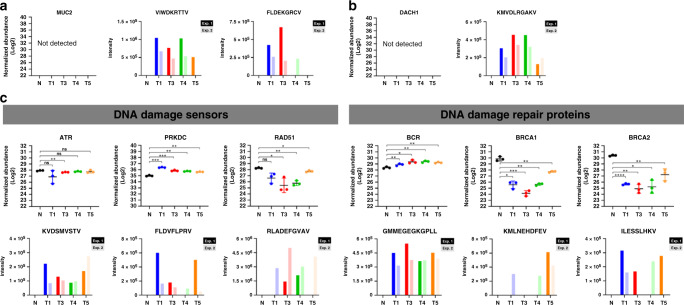
Clonal tumor HLA class I ligandomes over-represent peptides from DNA repair source proteins. **a** The MUC2 proteins were not detected in the deep proteome analysis (left), but two HLA peptide ligands derived from MUC2 degradation were predominantly presented by all CRC tumor organoid clones (right). For each peptide, normalized peptide intensities were summed from three replicated measurements by HCD (Exp1), or EThcD (Exp2), respectively. **b** The DACH1 protein was similarly not detected in the deep proteome analysis (left), but consistently presented on all tumor organoid clones, and consistently detected in both HCD and EThcD MS fragmentation methods. **c** Peptides derived from degradation of DNA damage sensors and repair proteins were prominently presented by CRC organoid clones as HLA peptides. Significant changes in protein abundance with respect to normal colon organoids were determined based on three technical replicates with a two-sided student’s *t* test: *p < 0.05; **p < 0.01. Data presented as mean values ± standard deviation. Plotting data tabulated in Source Data.

To rationalize the basis for tumor cells to present tumor-unique peptides, we looked into the source proteins of these ligands from each tumor clones, and compiled a master list of proteins that were reliably detected, in all six replicate mass spectrometry-based ligandome measurements per clone. In this stringently curated list of 356 source proteins, we observed several CRC prognostic markers and an over-representation in proteins involved in DNA damage sensing and repair. We hypothesized that proteins which are disadvantageous for propagation are more likely to be degraded in the tumor clones, and that in turn more peptides from these proteins may be presented on the cell surface by HLA class I molecules. In concurrence with this notion, HLA peptide ligands from MUC2 and DACH1 were reliably detected from all the CRC tumor clones analyzed (Fig. 5a, b), whereas we did not detect the proteins in the quantitative proteomics screen. This loss of MUC2 and DACH1 protein expression us not unique to our experimental model, but also broadly observed in CRC^34–36^. Along similar lines, HLA peptide ligands from numerous DNA damage sensing proteins (ATR, PRKDC, RAD51) and repair proteins (BCR, BRCA1, BRCA2) were also detected only in CRC organoid lines (Fig. 4c, Supplementary Fig. 5). We believe these peptides are likely products of active degradation by tumor cells, to prevent the induction of DNA damage response and hamper with DNA repair. Together, this would also support the acquisition of further genome instability, a documented hallmark of cancer^37^; since DNA damage remains “silent” despite severe chromosomal aberrations (Fig. 2b, c).

To strengthen our hypothesis in a reciprocal manner, we also performed source protein analysis on ligands that were presented only by normal colon organoids (Supplementary Fig. 6). ALDOA is a glycolytic enzyme observed to accumulate in CRC^38^, COPS6 is a subunit of the COP9 signalosome specifically required to drive CRC^39–41^, MCM2 functions as a DNA replication licensing factor needed to override once-per-cycle DNA replication in S-phase^42,43^ and SMARCA4 (BRG1) is a SWI/SNF component activating WNT and VEGF signaling to drive CRC^31,44,45^. While HLA peptides from these four proteins were reliably detected on normal colon organoids, these were consistently absent from the ligandome of all four CRC organoid lines, suggesting that these proteins may be functionally important in CRC and therefore preserved from degradation (Supplementary Fig. 6). Therefore, unique HLA peptide presentation on tumor organoids appears to be promoted by protein degradation events that favor oncogenesis and deter DNA damage remediation.

### Clone-specific ligandome characteristics and quantitative variations

**Fig. 6:**
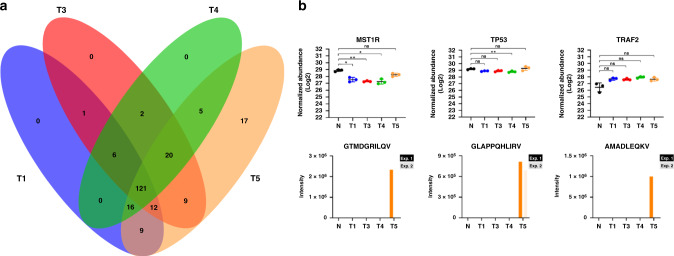
Clone specific ligandome characteristics. **a** Overlap of all predicted HLA ligands unique to at least one CRC organoid clone. Ligands were detected by either HCD (Exp 1) or EThcD (Exp 2) fragmentation. Most ligands not detected on normal colon organoids were shared by at least three tumor clones, except for 17 ligands unique to T5 only. **b** Protein abundance and peptide ligand abundance for MST1R, TP53, and TRAF2 from all organoid lines. Even though protein levels of MST1R, TRAF2, and TP53 were not significantly different between the four CRC tumor organoid lines, presentation of peptide ligands appear to be specific to only T5. Significant changes in protein abundance were determined with respect to normal colon organoids based on three technical replicates with a two-sided student’s *t* test: *p < 0.05; **p < 0.01. Data presented as mean values ± standard deviation. Plotting data tabulated in Source Data.

To further investigate ligandome heterogeneity between tumor clones, we focused on unique presentation by the CRC organoid clone T5. By plotting the overlap between tumor-specific HLA peptide ligands (from Fig. 4a), we observed that only T5 presented 17 clone-specific ligands that are not shared with the other three tumor organoid lines (Fig. 6a). HLA peptide ligands from MST1R, TP53, and TRAF2 were clearly differentially presented by T5, although proteome level trends of these proteins in all tumor clones were rather consistent with each other (Fig. 6b). To rule out differences in the peptide presentation pathway between the tumor clones, we also verified that components of the HLA presentation pathway^10^ are not prominently regulated in T5 compared to the other tumor clones (Supplementary Fig. 7). Therefore, unique degradation and presentation of these functionally relevant proteins hints at differential signaling and functional regulation specifically in T5, but not in the HLA processing machinery *per se*.

**Fig. 7:**
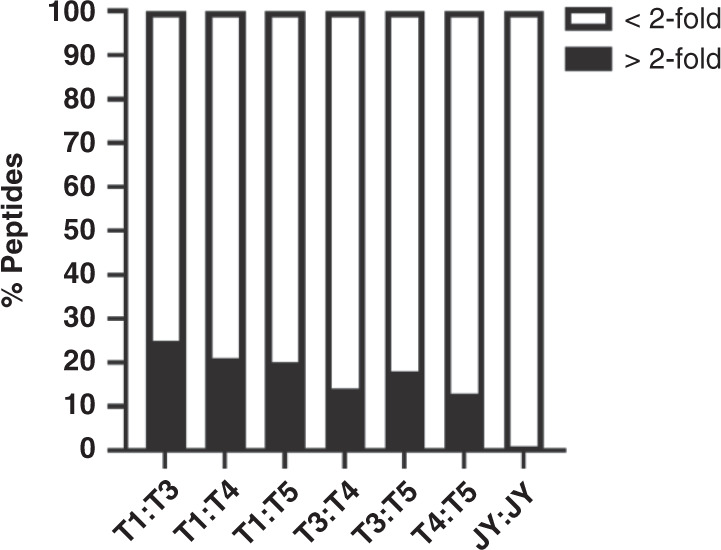
Clone-specific variations in peptide ligand presentation. Peptide ligand intensities from each tumor organoid clone were compared by retention time alignment and label-free quantification in a pair-wise manner. Between 15–25% of all ligands varied in intensity by more than 2-fold in each pairwise comparison (black). Quantitative variations by more than 2-fold were extremely few at <1%, between two independent preparations of JY cell line peptide ligands analyzed months apart with the same LC-MS settings, validating these quantitative ligand variations observed between tumor organoid clones were not of technical nature. Plotting data tabulated in Source Data.

Even though the HLA class I ligandome of the tumor clones consisted of largely shared peptide sequences (97% between CRC clones) significant quantitative variations in peptide abundance could still be observed. By pairwise comparison of HLA peptide intensities between different tumor clones, we observed that up to 15–25% of all the peptides detected could vary in intensity by more than 2-fold (Fig. 7). For instance, a SLINVGLISV peptide was 400 times more abundantly presented on T3 compared to T1. Therefore, this implies that one CRC organoid clone may present an HLA peptide ligand strongly, whereas another clone only weakly. We further verified that these intensity differences were not of technical nature, given that biological replicates of HLA ligandomes measured months apart did not vary by more than 1%. We believe such clone-to-clone variation in HLA peptide ligand presentation should be critically considered, especially in targeting residual disease after immunotherapy.

## Discussion

HLA class I peptide ligands presented abundantly, exclusively, and uniformly on the tumor surface provide ideal starting points to design personalized immunotherapy. In this work, we modeled single-cell level heterogeneity in CRC from single cells using patient-derived clonal organoids, and correlated clonal tumor proteomes with their respective HLA ligandomes.

The strategy presented here has various advantages. The three-dimensional spatial signaling in organoid culture recapitulates the gut (patho-)physiology more accurately than flat in vitro expansions. Parallel analysis of normal colon organoids from the same patient allowed us to directly assess specificity of proteome and ligandome signatures to CRC cells, in largely the same patient genetic background. In the background of low mutation load, we could subsequently survey the proteome and ligandome heterogeneity between individual clones largely without considering clone-specific genetic and protein-coding changes, to focus on studying the fundamental logic in cell-surface presentation. Since the proteome and ligandome are paired to each clone, steady-state protein abundance and HLA class I peptide ligand presentation could easily be correlated. Amplifying single cells using organoid technology also enables clone-specific proteome and ligandome signatures to be detected without signal averaging, unlike most of the tissue-based analyses reported to date. We believe our approach also adds sensitivity to identify a ligandome much larger than previously possible from patient-derived biopsies^46,47^.

Comparing between clonal proteomes of organoid-expanded tumor cells against normal colon organoids of the same patient, we observed that established cancer hallmarks were well-retained in every tumor organoid line, whereas these specific features were not present and also not acquired by the normal colon organoids during expansion, further verifying the validity of our experimental model. Whilst functional ontology of many proteome differences was conserved between tumor clones (e.g. mTOR), heterogeneity still exists in redundant pathways of epigenetic regulation and chromatin remodeling (e.g. KMTs and KATs). These observations also mirror the selective pressure in vivo, which evolves single cancer cells via multiple routes towards the same signaling outcome^48^.

More intriguingly, despite clear clustering of CRC organoid proteomes away from normal colon organoids, we observed that these protein-level changes are qualitatively buffered at the level of HLA peptide ligand presentation, such that tumor clones share up to 97% of ligandome overlap even with normal colon organoids. This enlightens on the perennial challenges in neo-antigen discovery^49,50^, where even in the context of a mutation-rich cancer, a large proportion of the tumor-surface HLA molecules is still occupied by peptides derived from routine protein turnover. More recently, neo-antigen discovery from non-coding regions have been attempted involving proteogenomics approaches and large computational efforts^51,52^. While these pipelines are streamlined to pick up the rare tumor-specific mutated antigen events, these approaches do not focus enough, during data analysis, on wild type ligands that are still over-presented from altered proteasome degradation and functional processing.

We propose, on the other hand, that tumor-specific HLA peptide ligand presentation could also arise as a byproduct, from the rational need to degrade tumor suppressors and proteins needed for DNA damage sensing and repair. Evidence supporting this “Achilles heel” is presented here in the paired whole-proteome and HLA class I peptide ligand analyses from multiple tumor clones, and is also logically concordant with the need to accumulate chromosomal lesions, which we observed in karyotypes. Even within the small tumor-specific ligand repertoire, and given the normally low abundance of BRCA proteins, we could still pick up tumor-specific BRCA peptides consistently across all the CRC clones analyzed, suggesting that this is likely reflecting an important molecular alteration in CRC. Since suppression of DNA damage sensing and repair is a common trait of many cancers, we believe the findings and rationale we distill here could also have strong impact on a variety of cancer and a range of immuno-therapeutic routes. For instance, potentially also in breast cancer with HER2 amplification but rapid turnover of HER2 by degradation^53^, where targeting HLA peptides derived from HER2 degradation may also be logical.

From a therapeutic point-of-view, HLA peptides with high tumor specificity and homogenously high tumor-surface loading are best candidates for further testing. While intense efforts have been channeled into predicting^51^ and detecting mutated and spliced ligands to boost tumor specificity^19,46,54,55^, relatively little has been studied regarding the presentation heterogeneity between clonal tumor populations. We believe the latter would be a strong determinant of therapeutic efficacy and residual disease, drawing upon lessons learned from chemo-resistance. We show here that by using single-cell amplified organoids from the same patient, that clone-specific ligandome signatures exist, and quantitative variations in clonal presentation are prevalent. In this respect, immunization with multiple peptides highly conserved in presentation, for instance BRCA peptides, may minimize the risk of immune escape.

## Methods

### Human material and informed consent

The organoid lines used in this study were derived from biopsies (provided by Departments of Surgery and Pathology of the Diakonessenhuis hospital, Utrecht, The Netherlands) of a CRC and adjacent healthy colon mucosal epithelium taken from colon tissue resected during a left hemicolectomy to remove a CRC from a female patient (71 years of age). Tissue collection was approved by the medical ethical committee (METC) of the Diakonessenhuis hospital, in agreement with the declaration of Helsinki and according to Dutch and European Union legislation. The samples were collected under METC protocol 12/093 HUB-Cancer following written informed consent at the Diakonessenhuis hospital Utrecht.

### Organoid generation and cultures

Colonic epithelial organoid lines were derived as previously described^56,57^. Briefly, crypts of the healthy portion of the colonic epithelium (at least 10 cm away from the tumor side) were isolated by digestion of the intestinal mucosa in chelation solution (5.6 mM Na_2_HPO_4_, 8.0 mM KH_2_PO_4_, 96.2 mM NaCl, 1.6 mM KCl, 43.4 mM Sucrose, and 54.9 mM D-Sorbitol, Sigma) supplemented with dithiotreitol (0.5 mM, Sigma) and EDTA (2 mM, in-house) for 30 min at 4 °C. Colonic crypts were subsequently plated in basement membrane extract (BME; Cultrex PC BME RGF type 2, Amsbio). Organoids were grown in human intestinal stem cell medium (HISCM), which is composed of Advanced Dulbecco’s modified Eagle medium/F12 supplemented with penicillin/streptomycin, 10 mM HEPES and Glutamax (all Gibco, Thermo Fischer Scientific) with 50% Wnt3a conditioned medium (in-house), 20% R-Spondin1 conditioned medium (in-house), 10% Noggin conditioned medium (in-house), 1x B27, 1.25mM N-acetyl cysteine, 10 mM nicotinamide, 50 ng/ml human EGF, 10 nM Gastrin, 500 nM A83-01, 3 µM SB202190, 10 nM prostaglandine E2 and 100 µg/ml Primocin (Invivogen). Tumor biopsies were digested into single cells using collagenase II (1 mg/ml, Gibco, Thermo Fischer Scientific), supplemented with hyaluronidase (10 µg/ml) and LY27632 (10 µM) for 30 min at 37 °C while shaking. Dissociated tumor cells were plated in BME and organoids were cultured in HICS minus Wnt conditioned medium and supplemented with 10 µM LY27632 at 37 °C.

### Clonal organoid derivation and amplification

To generate clonal tumor organoid lines, early passage tumor organoids were dissociated into single cells by TryPLE express (Thermo Fischer Scientific), washed and suspended in FACS buffer (PBS with 2 mM ETDA and 5% FCS). Prior to FACS purification, DAPI was added to the FACS buffer. HLA-A2^+^ single cells were sorted into separate wells of 96-wells plates containing 100 µl HISCM with 10 µM LY27632 and coated with BME. Sorted cells were then covered with 10 µl BME and placed in the incubator at 37 °C. LY27632 was added to the HISCM for the first week after sorting. Clonal tumor organoids were then expanded in HICS minus WNT conditioned medium.

### Whole-genome sequencing and somatic analysis of clonal organoid lines

Organoids were dissociated and DNA was isolated using the QiaSymphony DSP DNA Mini Kit (Qiagen; 937236). Libraries were prepared using the Illumina TruSeq DNA Nano Library Prep Kit (20015964). Paired-end sequencing of the organoid lines was performed (2 × 150 bp) on the generated libraries with 30× coverage using the Illumina HiSeq X Ten sequencing system at the Hartwig Medical Foundation. Somatic mutations were analyzed by the HMF somatic mutation workflow from https://github.com/hartwigmedical/pipeline which was installed the pipeline locally using GNU Guix with the recipe from https://github.com/UMCUGenetics/guix-additions. Full pipeline description is explained elsewhere^58^. Details and settings of all the tools can be found at their Github page. Briefly, sequence reads were mapped against human reference genome GRCh37 using Burrows-Wheeler Alignment (BWA-MEM) v0.7.5a. Subsequently, somatic single-base substitutions (SBSs) and small insertions and deletions (INDELS) were determined by providing the genotype and tumor (or organoid for in vitro analysis) sequencing data to Strelka v1.0.14 with adjustments as described in^58^.

### Organoid expansion and quantitative proteomics

Normal colon organoids and clonal tumor organoid lines were expanded in their respective media to about 5 × 10^8^ cells per organoid line. In the last medium change 3 days prior to harvest for peptidome analysis, the clonal tumor organoids lines received standard HISCM. On the day of harvest, organoids were removed from the 6-well culture plates using medium and P1000 pipettes and spun down for 8 min at 500 *g* with the brakes off. Cell pellets were then incubated with Cell Recovery Solution (Roche Diagnostics) for 30 min on ice to remove excess BME. Cells liberated from BME were then washed three times in excess PBS to remove any residual BME. After the last wash, all PBS was removed and the tube opening was quickly dried using a paper towel. Cell pellets were snap frozen in dry ice and stored at −80 °C.

Organoids were lysed by gentle vortexing in 8 M Urea in 50 mM ammonium bicarbonate supplemented with 50 µg/ml DNAse I (Sigma-Aldrich), 50 µg/ml RNAse A (Sigma-Aldrich) and 1× complete EDTA-free protease inhibitor cocktail (Roche Diagnostics). Subsequently, the lysate was cleared by centrifugation for 1 h at 18,000 *g* at 15 °C. Protein concentration was determined with the Bradford assay (Bio-Rad). For each sample, 20 µg of total protein was reduced, alkylated, and digested sequentially with Lys-C (1:100) and trypsin (1:75). For high-pH reversed-phase fractionation, peptide digests were loaded on C18 STAGE-tips in 200 mM ammonium formate at pH 10 and eluted into 5 fractions with 11–80% acetonitrile. All samples were dried by vacuum centrifugation and reconstituted in 10% formic acid prior to LC-MS/MS analyses. Per fraction, three technical replicates were measured by LC-MS/MS.

### Immuno-affinity purification

Organoids were lysed as described in Demmers et al, 2019^16^. In short, pHLA complexes were immunoprecipitated (nine IP equivalents per organoid clone) using 0.7 mg W6/32 antibody^59^ coupled to 175 µl protein A/G beads (Santa Cruz) from 35 mg organoid lysate. Antibodies were cross-linked to protein A/G beads to prevent co-elution. Incubation took place for approximately 16 h at 4 °C. After immunoprecipitation, the beads were washed with 40 ml cold PBS. pHLA complexes were subsequently eluted with 10% acetic acid. Peptide ligands were separated from HLA class I complexes using 10kD molecular weight cutoff filters (Millipore). The fraction containing HLA class I peptide ligands was dried by vacuum centrifugation and reconstituted in 10% formic acid prior to LC-MS/MS analyses. Per organoid clone, six technical replicates were measured of which three with EThcD fragmentation (Exp 1) and three with HCD fragmentation (Exp 2).

### Proteome LC-MS/MS analyses

The data were acquired with an UHPLC 1290 system (Agilent) coupled to a Q-Exactive HF mass spectrometer (Thermo Fischer Scientific). Peptides were trapped (Dr Maisch Reprosil C18, 3 µM, 2 cm × 100 µM) for 5 min in solvent A (0.1% formic acid in water) before being separated on an analytical column (Agilent Poroshell, EC-C18, 2.7 µM, 50 cm × 75 µM). Solvent B consisted of 0.1% formic acid in 80% acetonitrile. For high-pH reversed-phase samples (fraction 1) the gradient was as follows: first 5 min of trapping, followed by 85 min of gradient from 12 to 30% solvent B and, subsequently, 10 min of washing with 100% solvent B and 10 min of re-equilibration with 100% solvent A. For fraction 2 the gradient was from 15 to 32% solvent B. For fraction 3 the gradient was from 18 to 36% solvent B. For fraction 4 the gradient was from 20 to 38% solvent B and for fraction 5 from 22 to 44% solvent B. The mass spectrometer operated in data-dependent mode. Full scan MS spectra from *m/z* 375–1600 were acquired at a resolution of 60.000 to a target value of 3 × 10^6^ or a maximum injection time of 20 ms. The top 15 most intense precursors with a charge state of 2+ to 5+ were chosen for fragmentation. HCD fragmentation was performed at 27% normalized collision energy on selected precursors with 16 s dynamic exclusion at a 1.4 m/z isolation window after accumulation to 1 × 10^5^ ions or a maximum injection time of 50 ms. Tandem mass spectrometry (MS/MS) spectra were acquired at a resolution of 15.000.

### Ligandome LC-MS/MS analyses

The data were acquired with an UHPLC 1290 system (Agilent) coupled to an Orbitrap Fusion Lumos Tribrid (for EThcD fragmentation) mass spectrometer or a Q-Exactive HF-X (for HCD fragmentation) mass spectrometer (Thermo Fischer Scientific). Trapping and running conditions were similar as described above, with the exception of a 7–40% solvent B gradient. The mass spectrometer operated in data-dependent mode. Full scan MS spectra from *m/z* 400–650 were acquired at a resolution of 60.000 after accumulation to a target value of 4 × 10^5^ or a maximum injection time of 50 ms. Up to 3 (EThcD) or 15 (HCD) most intense precursors with a charge state of 2+ or 3+ starting at m/z 100 were chosen for fragmentation. EThcD fragmentation and HCD fragmentation were both performed at 35% normalized collision energy on selected precursors with 18 s (EThcD) or 16 s (HCD) dynamic exclusion after accumulation of 5 × 10^4^ ions or a maximum injection time of 250 ms. Tandem mass spectrometry (MS/MS) spectra were acquired at a resolution of 15.000.

### Proteome data analysis

Raw files were searched using MaxQuant version 1.5.3.30 and the Andromeda search engine against the human uniprot database (147854 entries, downloaded in January 2016). Enzyme specificity was set to trypsin and up to 2 missed cleavages were allowed. Cysteine carbamidomethylation was set as fixed modification. Methionine oxidation and N-terminal acetylation were set as variable modifications. The false discovery rate (FDR) was restricted to 1% in both protein and peptide identification. For quantitative comparisons, label-free quantification (LFQ) was performed with “match between runs” enabled. Data normalization, imputation, and statistics were performed with Perseus version 1.6.2.2. The data were visualized with Graphpad PRISM 8.

### Ligandome data analyses

Raw files were searched using Sequest HT in Proteome Discoverer 2.2 against the Swissprot human database (20258 entries, downloaded in Feb 2018) appended with the 20 most abundant FBS contaminants^60^. The search was set to unspecific with a minimum precursor mass of 797 Da to a maximum precursor mass of 1950 Da corresponding to peptides between 8 and 12 amino acids long. Identified peptides were filtered to a 1% FDR using the percolator algorithm, 5% peptide FDR and Xcorr >1. Cysteine cysteinylation and methionine oxidation were set as variable modifications. From the identified peptides, FBS contaminants were removed. Binding affinity of HLA class I peptide ligand was predicted using the NetMHC 4.0 pan algorithm^61^. The data were visualized with Graphpad PRISM 8.

### Reporting summary

Further information on research design is available in the Nature Research Reporting Summary linked to this article.

## Supplementary information

Supplementary Information

Reporting Summary

## Data Availability

The mass spectrometry proteomics and peptidomics data have been deposited to the ProteomeXchange Consortium via the PRIDE^62^ partner repository with the data set identifier PXD016582. The sequencing data of the CRC organoid lines have been deposited at the European Genome-phenome Archive (https://www.ebi.ac.uk/ega/studies/EGAS00001003366). Source data are provided with this paper.

## Source data

Source Data
