# Supplementary material for: Single-cell derived tumor organoids display diversity in HLA class I peptide presentation
Source: Nat Commun. 2020 Oct 21;11:5338. doi: 10.1038/s41467-020-19142-9 (PMC7577990; doi:10.1038/s41467-020-19142-9)
Supplement: Supplementary file 1 — Supplementary Information [file 41467_2020_19142_MOESM1_ESM.pdf]

Supplementary Information

Title

Single-cell derived tumor organoids display diversity in HLA class I peptide presentation

Authors

Laura C. Demmers[1, 2], Kai Kretzschmar[3, 4], Arne Van Hoeck[5], Yotam E. Bar-Epraïm[3, 4], Henk W. P. van den Toorn[1,2], Mandy Koomen[3,4], Gijs van Son[3,4], Joost van Gorp[6], Apollo Pronk[7], Niels Smakman[7], Edwin Cuppen[5,8], Hans Clevers[3,4,9], Albert J.R. Heck[1,2]*, Wei Wu[1,2]*

[1]Biomolecular Mass Spectrometry and Proteomics, Bijvoet Center for Biomolecular Research and Utrecht Institute for Pharmaceutical Sciences, Utrecht University, Padualaan 8, 3584 CH Utrecht, the Netherlands

[2]Netherlands Proteomics Centre, Padualaan 8, 3584 CH Utrecht, the Netherlands

[3]Oncode Institute, Hubrecht Institute, 3584 CT Utrecht, the Netherlands

[4]Hubrecht Institute, Royal Netherlands Academy of Arts and Sciences and University Medical Centre Utrecht, 3584 CT Utrecht, the Netherlands

[5]Center for Molecular Medicine and Oncode Institute, University Medical Center Utrecht, Universiteitsweg 100, 3584 CG Utrecht, The Netherlands.

[6]Department of Pathology, St. Antonius Hospital, 3543 AZ Utrecht, the Netherlands

[7]Department of Surgery, Diakonessenhuis Hospital, 3582 KE Utrecht, the Netherlands

[8]Hartwig Medical Foundation, 1098 XH Amsterdam, The Netherlands

[9]Princess Máxima Center for Pediatric Oncology, 3584 CS Utrecht, the Netherlands

*to whom correspondence should be addressed: w.wu1@uu.nl or a.j.r.heck@uu.nl

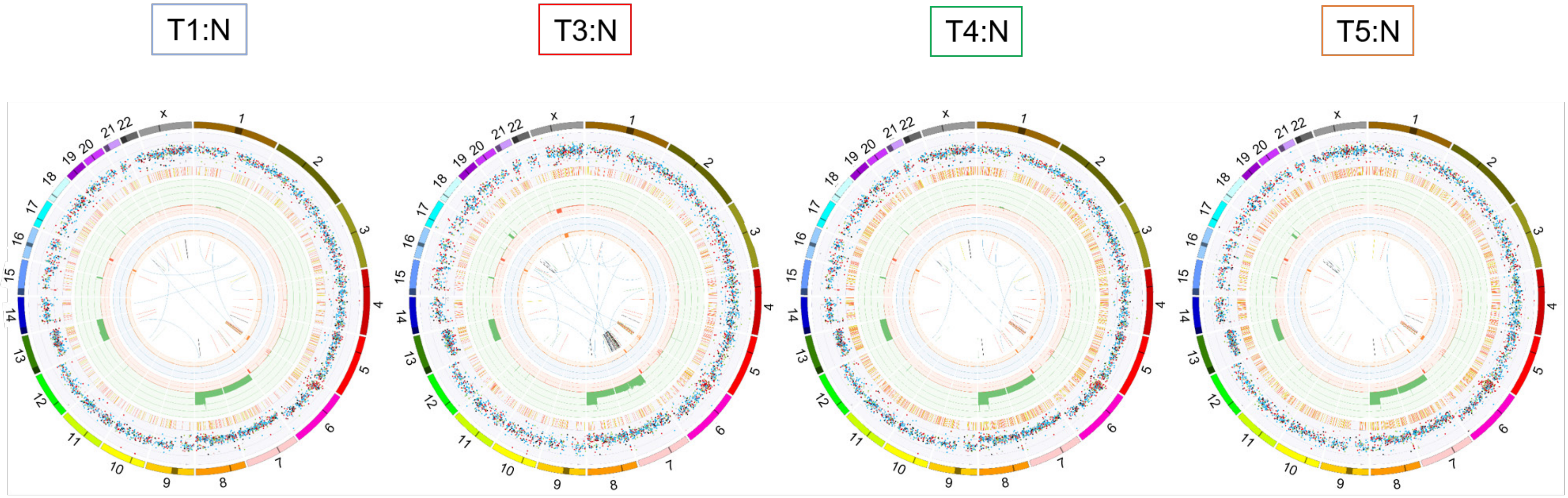

Supplementary Figure 1: Circoplots of genetic alterations detected in the single-cell derived clonal colorectal organoids. The outermost circle demarcate chromosomes. The next concentric ring shows the somatic variants (including exon, intron and intergenic regions) with respect to N. Somatic variants are further divided into an outer ring of substitutions and an inner ring of INDELs. Each dot represents a single somatic variant scaled to 100% by its allele frequency score. Point mutations are colored to the type of base change (e.g. C>T/G>A in red) and are in concordance with colors used previously to describe mutational signatures[63]. INDELs are colored yellow and red for insertions and deletions respectively. The third ring shows copy number changes adjusted against observed tumor purity. Copy number loss and gain are indicated in red and green respectively. The scale ranges from 0 (complete loss) to 6 (high level gains). The fourth ring represents the observed 'minor allele copy numbers' across the chromosome. The range of this chart is from 0 to 3. The expected normal minor allele copy number is 1 and anything below 1 is shown as a loss (orange) and represents a LOH event. Minor allele copy numbers above 1 (blue) indicate amplification events of both A and B alleles at the indicated locations. The innermost circle displays the observed structural variants within or between the chromosomes. Translocations are indicated in blue, deletions in red, insertions in yellow, tandem duplications in green and inversions in black.

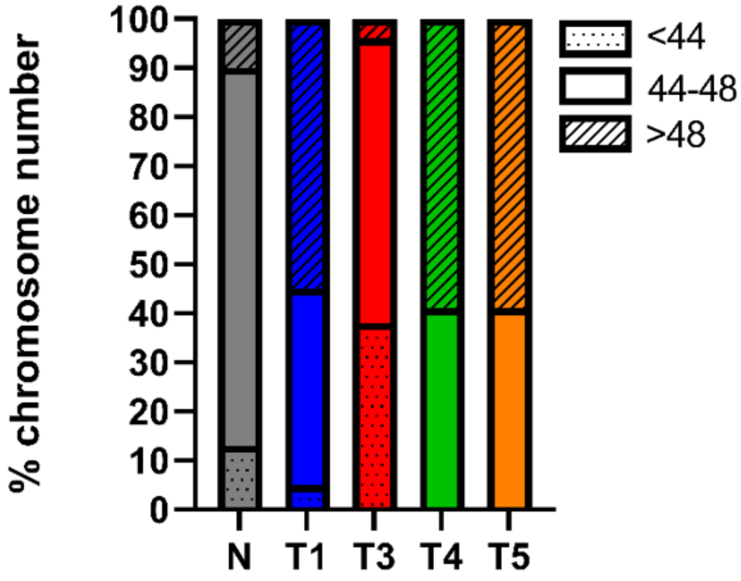

Supplementary Figure 2: Single-cell derived clonal colorectal organoids karyotyping. Percentage of cells with aberrant number of chromosomes. CRC tumor organoids were evidently enriched for aneuploid cells. Karyotyping was performed as described in Drost et al, 2015 [64] on organoid lines cryopreserved at the time of collection for proteomics measurements, subsequently, recovered and expanded for karyotyping. Per organoid line, between 30 and 46 metaphase spreads were counted. Plotting data tabulated in Source Data.

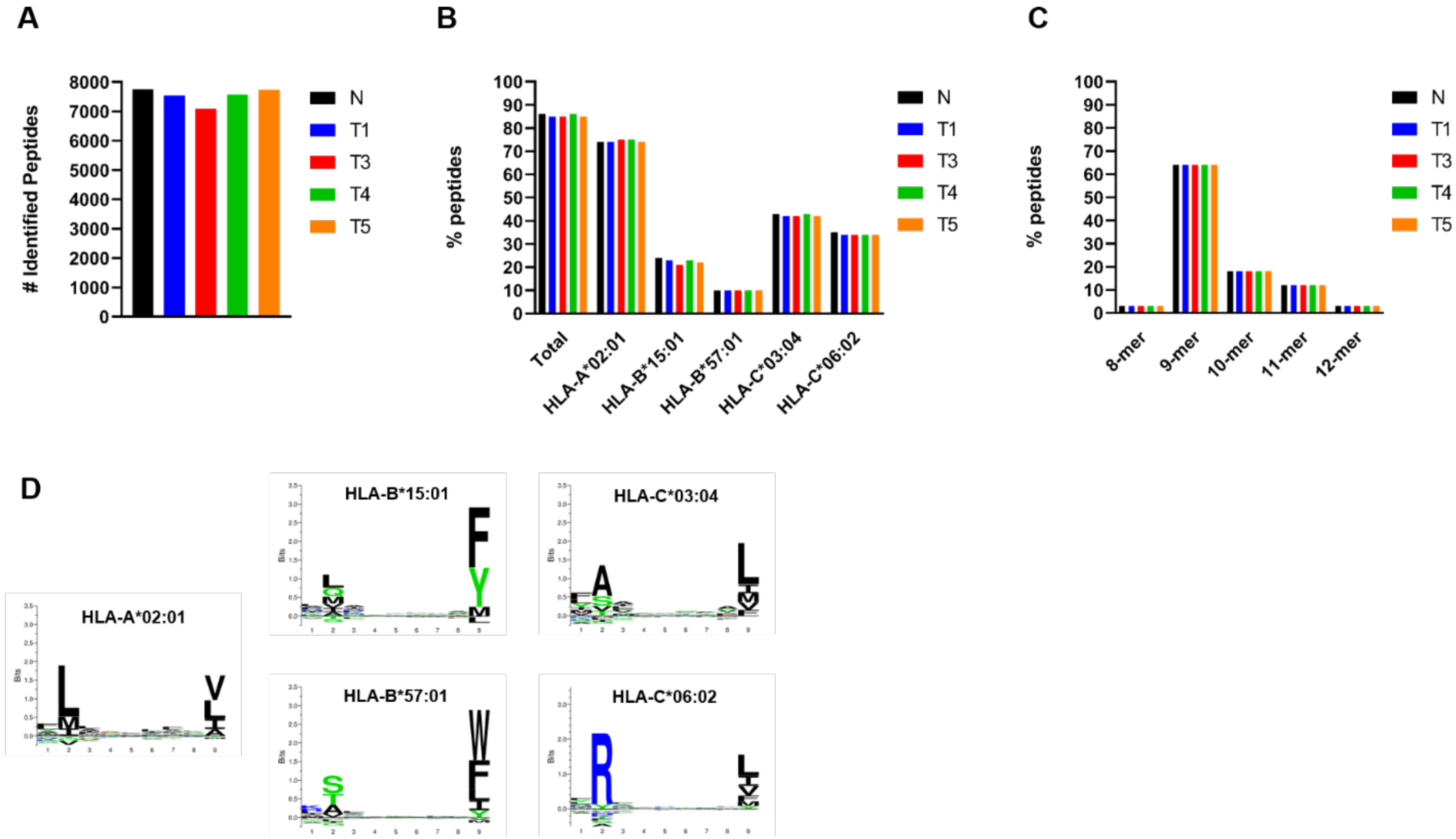

Supplementary Figure 3: HLA class I peptide ligand characteristics. A) Number of unique peptides identified per organoid clone (N = 7750, T1 = 7541, T3 = 7087, T4 = 7571, T5 = 7741). B) Assignment of all identified peptides to the patient's HLA alleles. C) Length distribution of identified HLA class I peptide ligands. D) Theoretical binding motifs of the HLA class I peptide ligands from the patient's HLA alleles. Plotting data tabulated in Source Data.

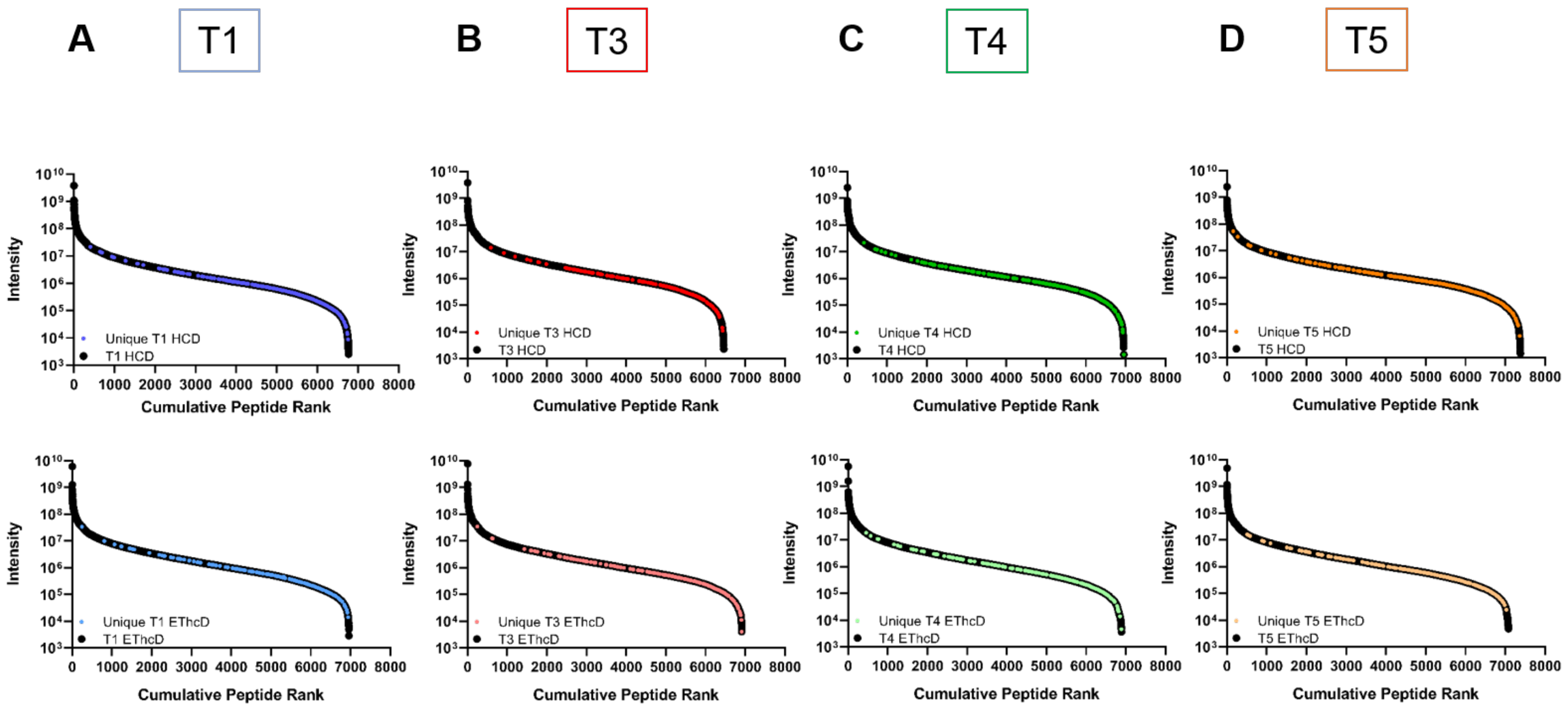

Supplementary Figure 4: Unique peptide ligand intensity distribution per tumor organoid. A) Peptide intensity plotted against the cumulative peptide rank for all T1 peptides (black) and T1 unique peptides (HCD in blue, EThcD in light blue). B) Peptide intensity plotted against the cumulative peptide rank for all T3 peptides (black) and T3 unique peptides (HCD in red, EThcD in light red). C) Peptide intensity plotted against the cumulative peptide rank for all T4 peptides (black) and T4 unique peptides (HCD in green, EThcD in light green). D) Peptide intensity plotted against the cumulative peptide rank for all T5 peptides (black) and T5 unique peptides (HCD in orange, EThcD in light orange). Plotting data tabulated in Source Data.

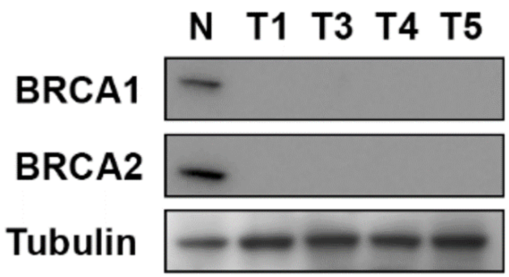

Supplementary Figure 5: BRCA1 and BRCA2 western blot. Both BRCA1 (SC-6954, dilution 1:500) and BRCA2 (SC-293185, dilution 1:500) were not detectable in all four tumor organoid clones, in agreement with the down-regulation observed in fractionated organoid proteomes (Figure 5C). Tubulin was used as loading control. Raw images in Source Data.

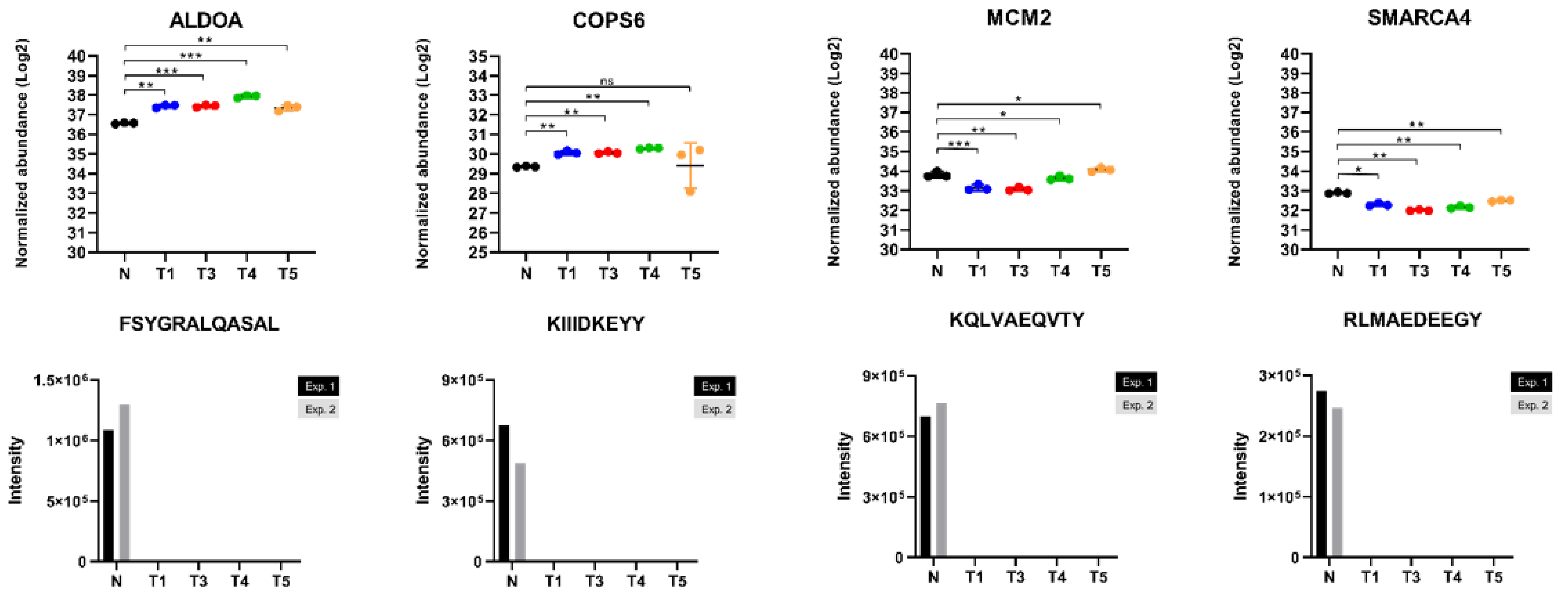

Supplementary Figure 6: Protein abundance and HLA peptide ligand abundance for ALDOA, COPS6, MCM2 and SMARCA4 in all analyzed organoid clones. Top panels: Normalized abundance of the proteins ALDOA, COPS6, MCM2 and SMARCA4, as measured by label-free quantitative proteomics. Bottom panels: HLA peptides originating from ALDOA, COPS6, MCM2 and SMARCA4 were reproducibly detected on normal colon organoids by both HCD (Exp 1) and EThcD (Exp 2) MS fragmentation modes, but not detected in any of the four CRC tumor clones by either fragmentation mode. Significant changes in protein abundance were determined with respect to normal colon organoids based on three technical replicates with a two-sided student's *t*-test: $p < 0.05$ (*); $p<0.01$(**). Data presented as mean values ± standard deviation. Plotting data tabulated in Source Data.

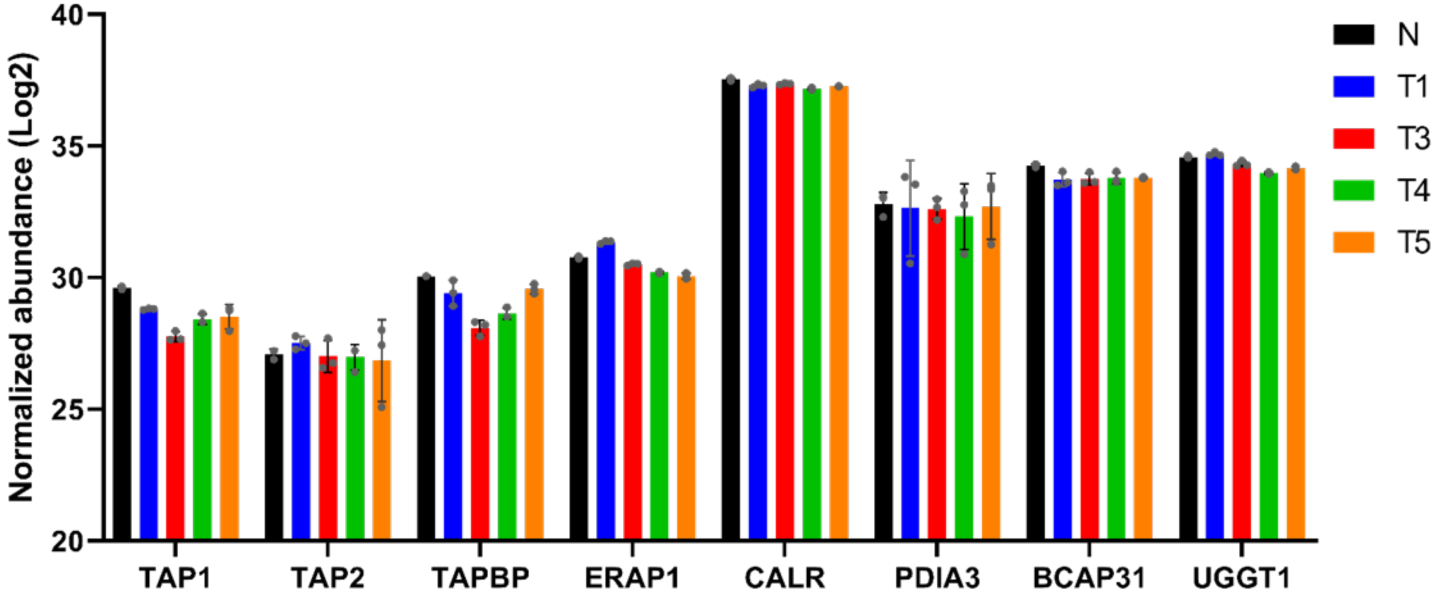

Supplementary Figure 7: Protein abundance of protein involved in HLA class I processing in all analyzed organoid clones. n = 3 technical replicates. Data presented as mean values ± standard deviation. Plotting data tabulated in Source Data.
