# Supplementary material for: Single-cell derived tumor organoids display diversity in HLA class I peptide presentation
Source: Nat Commun. 2020 Oct 21;11:5338. doi: 10.1038/s41467-020-19142-9 (PMC7577990; doi:10.1038/s41467-020-19142-9)
Supplement: Supplementary file 2 — Reporting Summary [file 41467_2020_19142_MOESM2_ESM.pdf]

nature research

Corresponding author(s): Wei Wu

Last updated by author(s): Sep 21, 2020

Reporting Summary

Nature Research wishes to improve the reproducibility of the work that we publish. This form provides structure for consistency and transparency in reporting. For further information on Nature Research policies, see our Editorial Policies and the Editorial Policy Checklist.

Statistics

For all statistical analyses, confirm that the following items are present in the figure legend, table legend, main text, or Methods section.

n/a	Confirmed	
☐	☒	The exact sample size (n) for each experimental group/condition, given as a discrete number and unit of measurement
☐	☒	A statement on whether measurements were taken from distinct samples or whether the same sample was measured repeatedly
☐	☒	The statistical test(s) used AND whether they are one- or two-sided<br>*Only common tests should be described solely by name; describe more complex techniques in the Methods section.*
☒	☐	A description of all covariates tested
☒	☐	A description of any assumptions or corrections, such as tests of normality and adjustment for multiple comparisons
☐	☒	A full description of the statistical parameters including central tendency (e.g. means) or other basic estimates (e.g. regression coefficient) AND variation (e.g. standard deviation) or associated estimates of uncertainty (e.g. confidence intervals)
☐	☒	For null hypothesis testing, the test statistic (e.g. F, t, r) with confidence intervals, effect sizes, degrees of freedom and P value noted<br>*Give P values as exact values whenever suitable.*
☒	☐	For Bayesian analysis, information on the choice of priors and Markov chain Monte Carlo settings
☒	☐	For hierarchical and complex designs, identification of the appropriate level for tests and full reporting of outcomes
☒	☐	Estimates of effect sizes (e.g. Cohen's d, Pearson's r), indicating how they were calculated

Our web collection on statistics for biologists contains articles on many of the points above.

Software and code

Policy information about availability of computer code

Data collection	Orbitrap Tune 3.3 (Thermo Scientific); XCalibur (version n/a)
Data analysis	MaxQuant version 1.5.3.30; Perseus 1.6.2.2; Graphpad PRISM 8; Proteome Discoverer2.2; NetMHC 4.0

For manuscripts utilizing custom algorithms or software that are central to the research but not yet described in published literature, software must be made available to editors and reviewers. We strongly encourage code deposition in a community repository (e.g. GitHub). See the Nature Research guidelines for submitting code & software for further information.

Data

Policy information about availability of data

All manuscripts must include a data availability statement. This statement should provide the following information, where applicable:
- Accession codes, unique identifiers, or web links for publicly available datasets
- A list of figures that have associated raw data
- A description of any restrictions on data availability

The mass spectrometry proteomics and peptidomics data have been deposited to the ProteomeXchange Consortium via the PRIDE partner repository with the data set identifier PXD016582, and will be available for public access without password after manuscript acceptance. The sequencing data of the CRC organoid lines have been deposited at the European Genome-phenome Archive (https://www.ebi.ac.uk/ega/studies/) under accession number EGAS00001003366.

Field-specific reporting

Please select the one below that is the best fit for your research. If you are not sure, read the appropriate sections before making your selection.

- [x] Life sciences
- [ ] Behavioural & social sciences
- [ ] Ecological, evolutionary & environmental sciences

For a reference copy of the document with all sections, see nature.com/documents/nr-reporting-summary-flat.pdf

Life sciences study design

All studies must disclose on these points even when the disclosure is negative.

Sample size	This work features comparison between organoid lines generated from different cells of a single patient, and is aimed at documenting intra-patient variability. Hence n size is not a valid consideration, since all comparisons are made within the same patient. Instead we maximised the number of tumor clones we can handle in one batch, which is 1 normal and 4 tumor clones. These experiments require large amounts of starting organoids, and parallel processing, we were constrained by batch feasibility, manpower and cost.
Data exclusions	No data was excluded.
Replication	This work features comparison between organoid lines generated from different cells of a single patient. Given the highly personalised way these organoid lines were derived, replication from the same donor is not possible after the first surgical ressection where tumor material was isolated. The process of organoid generation is also very time consuming, and not within the timeline possible in this manuscript to replicate in another organoid donor. Most importantly, replication is not a valid consideration in our opinion, since all comparisons are made within the same patient in the study design to focus on personalised changed in HLA peptide presentation. In all comparisons, three technical replicates were used for statistics.
Randomization	Sample order was randomised during measurement on the mass spectrometer
Blinding	Since we work with resected material from a patient, tumor/normal cell status is clearly documented in every stage of the study. Particularly in organoid generation, the factors required for organoid establishment differ between normal and tumor lines. Blinding is thus not possible at the operator level.

Reporting for specific materials, systems and methods

We require information from authors about some types of materials, experimental systems and methods used in many studies. Here, indicate whether each material, system or method listed is relevant to your study. If you are not sure if a list item applies to your research, read the appropriate section before selecting a response.

Materials & experimental systems

n/a	Involved in the study	
☐	☒	Antibodies
☒	☐	Eukaryotic cell lines
☒	☐	Palaeontology and archaeology
☒	☐	Animals and other organisms
☒	☐	Human research participants
☒	☐	Clinical data
☒	☐	Dual use research of concern

Methods

n/a	Involved in the study	
☒	☐	ChIP-seq
☒	☐	Flow cytometry
☒	☐	MRI-based neuroimaging

Antibodies

Antibodies used	BRCA-1 antibody (SC-6954, clone D-9, lot #2719, Santa Cruz). BRCA-2 antibody (SC-293185, clone 3D12, lot #B1120, Santa-Cruz). W6/32 antibody kindly provided by Dr. Stefan Stevanović, hence catalog and lot number are not applicable.
Validation	sc-6954: PMID: 31270457, PMID: 31570788, PMID: 30886146, + ~270 on https://www.scbt.com/p/brca1-antibody-d-9<br>SC-293185: PMID: 30297739, PMID: 30171044, PMID: 31209201, PMCID: PMC7285565 DOI: 10.1186/s13046-020-01597-9<br>W6/32: PMID: 30784271, PMID: 29476514, PMID: 31092913
